# Research on control strategy of vehicle stability based on dynamic stable region regression analysis

**DOI:** 10.3389/fnbot.2023.1149201

**Published:** 2023-03-13

**Authors:** Zhaoyong Liu, Yihang Li, Weijun Li, Zefan Li, Haosen Zhang, Xiaoqiang Tan, Guangqiang Wu

**Affiliations:** ^1^School of Automotive Studies, Tongji University, Shanghai, China; ^2^Global Technology Co., Ltd., Nantong, China

**Keywords:** vehicle phase plane, stability judgement, SVR, stability control, LTV-MPC, AEB

## Abstract

The intervention time of stability control system is determined by stability judgment, which is the basis of vehicle stability control. According to the different working conditions of the vehicle, we construct the phase plane of the vehicle's sideslip angle and sideslip angular velocity, and establish the sample dataset of the stable region of the different phase planes. To reduce the complexity of phase plane stable region division and avoid large amount of data, we established the support vector regression (SVR) model, and realized the automatic regression of dynamic stable region. The testing of the test set shows that the model established in this paper has strong generalization ability. We designed a direct yaw-moment control (DYC) stability controller based on linear time-varying model predictive control (LTV-MPC). The influence of key factors such as centroid position and road adhesion coefficient on the stable region is analyzed through phase diagram. The effectiveness of the stability judgment and control algorithm is verified by simulation tests.

## 1. Introduction

Active safety technology has increasingly become one of the key research fields of the automotive industry. The stability of the vehicle indicates the safety of the vehicle driving, and vehicle stability control is the basis for the implementation of active safety technology (Lai et al., 2021). The judgment of vehicle stability determines the intervention and exit time of the control system, which is an extremely critical part of stability control (Chen et al., 2020).

There are two mainstream methods of vehicle stability judgment. The first is to use the stability criterion, such as Lyapunov criterion, in control theory to conduct stability judgment based on a multi-DOF model of the vehicle or tire (Zhenyong, 2006; Yang et al., 2009; Vignati et al., 2017). The second is to use the phase plane to judge the stability of the vehicle, which is very intuitive. It is an important research method of vehicle stability judgment.

The vehicle stability judgment methods based on the phase plane can be divided into two main types: the sideslip angle—yaw rate phase plane method and the sideslip angle—sideslip angular velocity phase plane method. Because the sideslip angle—yaw rate phase plane method cannot accurately judge the vehicle stability under the unstable conditions such as pure sideslip with small yaw rate fluctuation, while the sideslip angle—sideslip angular velocity phase plane method does not have this problem, so the latter is more widely used (Inagaki et al., 1995).

The sideslip angle-sideslip angular velocity phase plane method was originally proposed by Inagaki et al. (1995) and Yamamoto et al. (1995). They use the “double-line method” to distinguish vehicle stability. Two straight lines passing through the saddle point are determined in the sideslip angle-sideslip angular velocity phase plane. The region surrounded by these two straight lines is considered as the stable region in the phase plane, but the stable region still contains many unstable trajectories far from the equilibrium point. Taeyoung and Kyongsu (2006) proposed to determine a rhombic region in the sideslip angle-sideslip angular velocity phase plane as the stable region of the vehicle. The four vertices of the rhomb fall on the two coordinate axes, and the vehicle stability control with variable threshold is achieved by setting the relaxation factor. The experimental results show that the stability control scheme has good performance, but more parameters will be introduced at the same time, which leads to difficulties in dividing stable region. Von Vietinghoff et al. (2008) verified the work of Taeyoung and Kyongsu (2006) by simulation and found that the rhombic method may not be able to determine the upper and lower endpoints. Yu et al. (2015) introduced the stable region determined by the yaw rate method based on the double-line method, reduced the unstable operating conditions in the stable region obtained by the double-line method, and established a database of stable region at different vehicle speeds, road adhesion coefficients and front wheel angles. Liu et al. (2014) proposed an improved five-eigenvalue rhombus stable region determination scheme, and established a stability region database for different vehicle speeds, road adhesion coefficients and front wheel angles through simulation. During the simulation process, the table can be checked according to vehicle state parameters to judge vehicle stability. In summary, most of the existing documents have considered the effect of real-time vehicle speed, adhesion coefficient and front wheel angle on the phase plane. In fact, due to the uncertainty of the mass and position of the load and passengers, the mass and centroid position of the vehicle will change. These changes will lead to great changes in vehicle performance, such as braking performance, acceleration performance and anti-roll performance. Therefore, it is necessary to consider the change of centroid position when plotting the vehicle phase plane.

At present, in the study of using the phase plane method to determine the driving stability of vehicles, most of them use the method of establishing databases and looking up tables to determine the stable region under different working conditions. This scheme can meet the accuracy requirements, but when the vehicle parameters change, the database needs to be reconstructed, resulting in high time and space complexity and poor practicability. With the rapid development of data transmission and artificial intelligence technology, machine learning algorithms are widely used in various disciplines to solve various classification and regression problems. The division of stable regions of vehicles in different states is also a regression prediction problem of data feature extraction, which can be solved by machine learning.

In this paper, based on the traditional double-line method, we proposed an improved double-line method for stable region division considering the limit value of the sideslip angular velocity. Then, we designed the SVR vehicle stable region regression model with a small dataset, which can make reasonable speculation on the stable region of the vehicle. In addition, we constructed a DYC controller to verify its feasibility and superiority.

**Figure 1:**
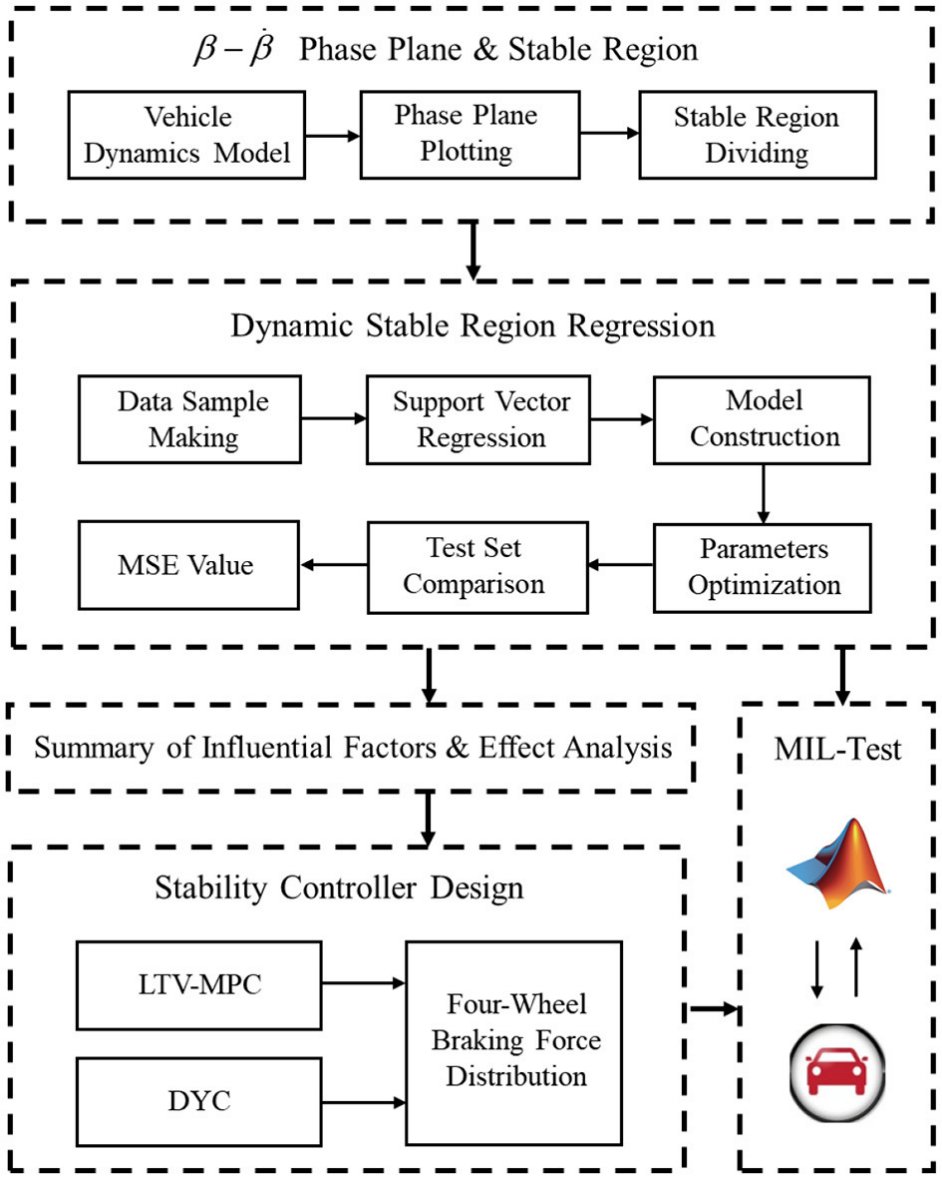
Thesis structure.

The structure of this paper is as follows. Section 1 is the introduction of the background. Section 2 introduces the process of vehicle $\beta -\dot{\beta }$ phase plane plotting and stable region dividing. Section 3 introduces the dynamic stable region regression model, including data sample making, model construction, parameters optimization and test set comparison. Section 4 introduces the summary of influential factors and effect analysis. Section 5 introduces the design of the stability controller and simulation test scheme, and analyzes the simulation results. Section Conclusion is the conclusion of the paper. The architecture of this paper is shown in Figure 1.

## 2. $\beta -\dot{\beta }$ phase plane establishment and stable region division

### 2.1. Vehicle dynamics modeling

**Figure 2:**
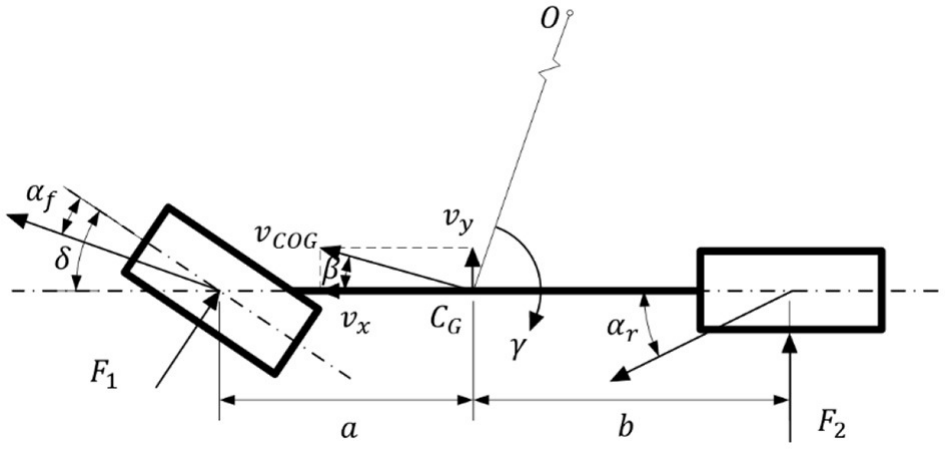
Vehicle dynamics model.

As shown in Figure 2, this paper carries out vehicle driving stability research based on a 2-DOF nonlinear monorail model, where β denotes the sideslip angle, δ denotes the front wheel angle, α_*f*_ denotes the front wheel slip angle, α_*r*_ denotes the rear wheel slip angle, *v*_*COG*_ denotes the velocity at the centroid of the vehicle, γ denotes the yaw rate, *C*_*G*_ denotes the centroid of the vehicle, *O* denotes the instantaneous center of the steering motion of the vehicle at this moment, *a* denotes the distance from the centroid to the front axis, *b* denotes the distance from the centroid to the rear axis, *F*_1_ indicates the lateral force on the front axle and *F*_2_ indicates the lateral force on the rear axle.

According to the 2-DOF monorail model of the vehicle shown in Figure 2, the kinematics equation of the whole vehicle is derived from Newton's law. As shown in (1), where *m* denotes the mass of the whole vehicle, *v*_*x*_ denotes the component of the vehicle velocity in the X-axis direction, *I*_*z*_ denotes the rotational inertia of the whole vehicle around the Z-axis.


(1) $$\{\begin{array}{c} \dot{\beta }=\frac{cos\text{β}}{mv_{x}}\left[F_{1}cos\delta +F_{2}\right]-\frac{sin\text{β}}{mv_{x}}F_{1}sin\delta -\gamma \\ \ddot{\beta }=-\frac{sin\text{β}}{mv_{x}}\left[F_{1}cos\delta +F_{2}\right]-\frac{cos\text{β}}{mv_{x}}F_{1}sin\delta -\dot{\gamma }\\ \;\dot{\gamma }=\frac{F_{1}cos\delta a-F_{2}b}{I_{z}} \end{array}$$

### 2.2. Phase plane plotting

**Figure 3:**
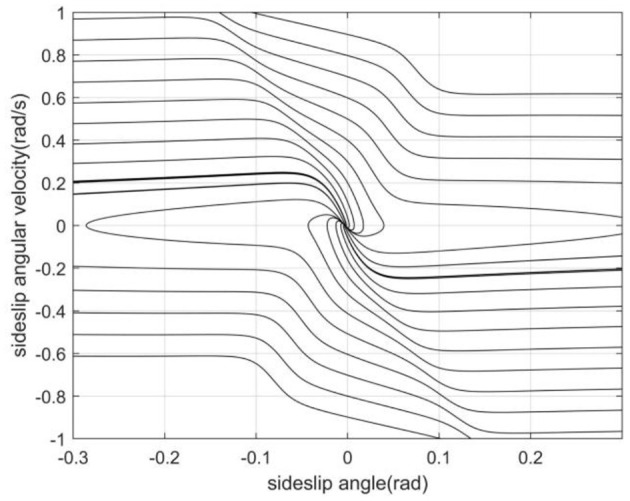
Phase plane trajectory with road adhesion coefficient 0.2, longitudinal speed 11 m/s and front wheel angle 0°.

In this paper, the lateral forces on the front and rear axles of the vehicle under different road adhesion coefficient, centroid position, front wheel angle and speed are obtained through simulation tests (Zha et al., 2021). According to the formula (1), the changes of the sideslip angle and the sideslip angular velocity under different working conditions can be calculated. Using the parameters in Table 1, the phase trajectories of sideslip angle and sideslip angular velocity can be drawn (Li et al., 2014), as shown in Figure 3.

**Table 1:** Partial parameter setting of the vehicle model.

**Parameters**	**Values**	**Units**
Mass of the vehicle *m*	1, 690	*kg*
Rotational inertia around the Z-axis *I*_*z*_	4, 192	*kg*/*m*^2^
Wheel base *L*	2.776	*m*
Distance between roll axis and centroid *h*_*r*_	0.50	*m*

### 2.3. Stable region dividing

**Figure 4:**
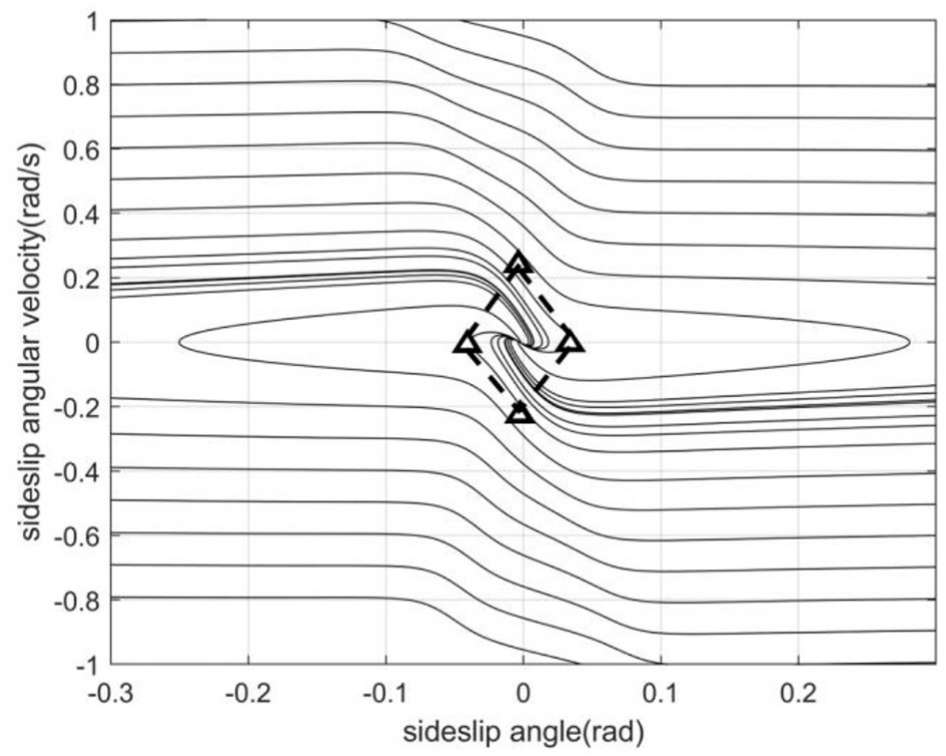
Phase plane stable region.

This section proposes a method for dividing the stable region in the $\beta -\dot{\beta }$ phase plane based on the improved double-line method. We establish the coordinate axis with the balance point as the center, its intersection with the two parallel lines on the phase plane which are tangent to the phase trajectory are the boundary values of the vehicle stability region, and their intersection points with the transverse axis of the equilibrium point are called saddle points, which characterize the limit value of the sideslip angle. The left and right boundary values of the quadrilateral stable region are the left saddle point and the right saddle point respectively. The upper and lower boundary values are composed of two intersections of the double parallel lines and the vertical axis of the equilibrium point, as shown in Figure 4. Since the phase trajectory in this region always extends in the direction of decreasing the absolute value of the sideslip angle, controlling the centroid sideslip angle in the phase plane stable region can effectively maintain the lateral stability of the vehicle (Zhang et al., 2011). The phase plane represents the relationship between the sideslip angle and the sideslip angular velocity. Its phase trajectory varies according to the changes of the centroid position, road adhesion coefficient, vehicle speed and front wheel angle, etc.

From the above, the four boundaries and equilibrium points of the regression model output determine the stable region. The specific methods will be described in the next section.

## 3. Dynamic stable region regression and stability judgment

### 3.1. Sample making

SVR machine is widely used to solve data regression problems because of its good predictive property for small dataset and its robustness to abnormal data (Zhang et al., 2019). In this paper, we will use SVR to realize the regression of phase plane stable region. Firstly, the samples of dataset are made according to the improved double-line method, i.e., the phase plane is artificially divided and the information of the division of stable region is recorded. To study the effects of centroid position, vehicle speed, road adhesion coefficient and front wheel angle on the phase plane trajectory, set the working condition parameters as shown in Table 2.

**Table 2:** Working condition parameters setting.

**Descriptions**	**Symbols**	**Values**	**Units**
Ratio of distance from center of mass to front axle and wheel base	α/L	[0.4, 0.4881]	–
Vehicle speed	v	[11, 20, 30]	m/s
Road adhesion coefficient	μ	[0.2, 0.6, 1.0]	–
Front Wheel Angle	δ	[0, 10, 20, 30]	rad

**Figure 5:**
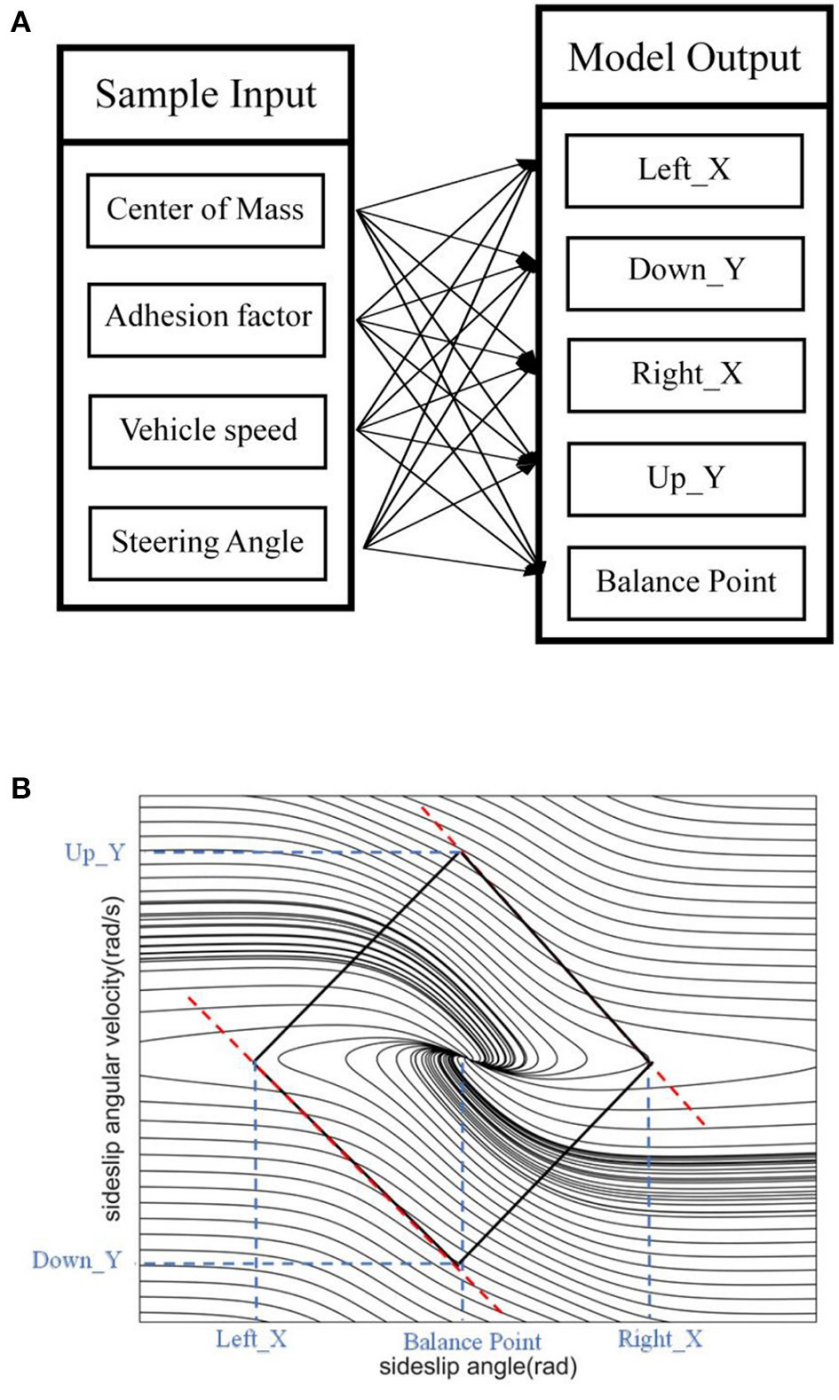
SVR model. **(A)** Model input-output mapping relationship. **(B)** Model predicted value output information.

In this paper, five MISO SVR models are established based on data sets, as shown in Figure 5A. The input and output of the model have the following mapping relationship. The model output includes three sideslip angle boundary predictions and two sideslip angular velocity boundary predictions, and each output is shown in Figure 5B. The data set is divided into training set and test set according to the ratio of 8:2. The training set is used to train the SVR model, and the test set is used to evaluate its performance, as shown below.

### 3.2. Model structure

For linearly divisible SVM problems (Huang et al., 2014), a convex optimization problem needs to be solved by the maximum interval algorithm: minimizing a linear inequality constrained quadratic function. Given linearly differentiable training samples whose number is *l*, the optimization problem is solved as follows (Sun et al., 2008).


(2) $$S=\;(\left(x_{1},y_{1}\right),\ldots ,\;(x_{l},y_{l}))$$

(3) $$\begin{array}{l} {\text{minimise}}_{w,b}\langle w\;\cdot \;w\rangle ,\text{ subject to }y_{i}\left(\langle w\;\cdot \;x_{i}\rangle +b\right)\\ \geq 1\;i=1,\ldots ,l \end{array}$$

The solution process requires the transformation of the above optimization problem using Lagrange function, from the minimax problem to the corresponding duality problem, and then substituted back into the original equation to obtain the following objective function, which is further solved by sequential minimal optimization (SMO) algorithm.


(4) $$\begin{array}{l} L\left(w,b,a\right)=\frac{1}{2}\langle w\;\cdot \;w\rangle -\sum_{i=1}^{l}\alpha_{i}\left(y_{i}\left(\langle w\;\cdot \;x_{i}\rangle +b\right)-1\right)\\ \;=\sum_{i=1}^{l}\alpha_{i}-\frac{1}{2}\sum_{i,j=1}^{l}y_{i}y_{j}\alpha_{i}\alpha_{j}\langle x_{i}\;\cdot \;x_{j}\;\rangle \end{array}$$

where α_*i*_ ≥ 0 are the Lagrange multipliers.

However, the hard margin classifier mentioned above cannot be used in many real-world problems. If the experimental data are noisy, a soft margin classifier is used to allow the model to tolerate noise and outliers, thus taking more training points into account, which is a class of problems called linear SVM. We introduce a margin relaxation factor, which allows the formula to violate the margin constraint to some extent, when the optimization problem becomes:


(5) $$\begin{array}{r} {\text{minimise}}_{\xi ,w,b}\langle w\;\cdot \;w\rangle +C\sum_{i=1}^{l}\xi_{i}^{2},\text{ subject to }y_{i}\left(\langle w\;\cdot \;x_{i}\rangle +b\right)\\ \;\geq 1-\xi_{i}\;i=1,\ldots ,l\;\xi_{i}\geq 0 \end{array}$$

**Figure 6:**
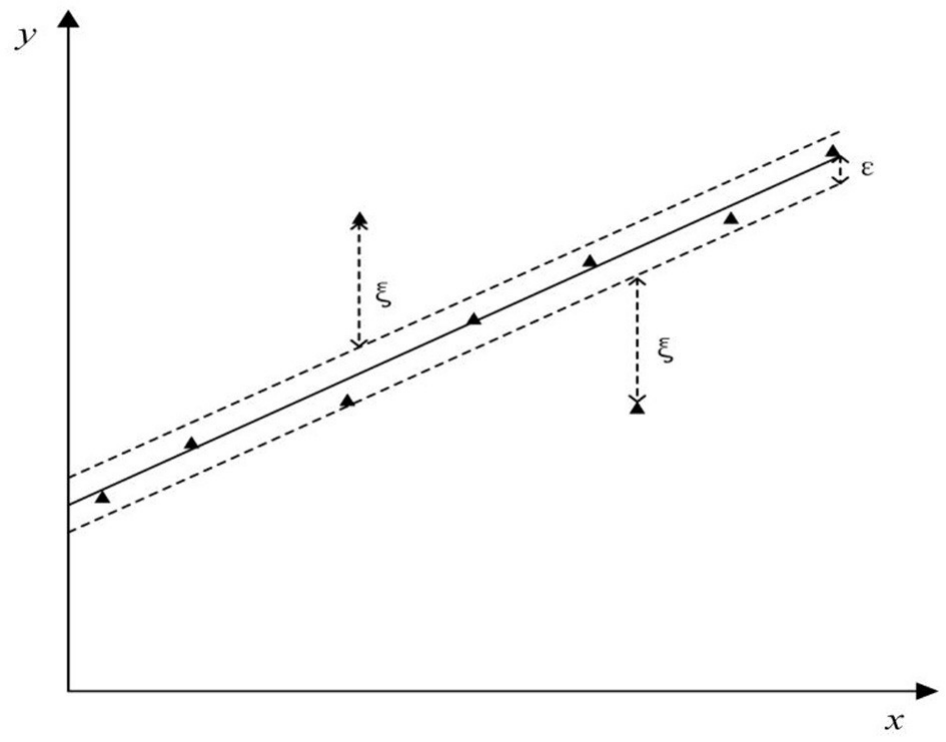
ε−*SVR* regression error bound.

The SVR, which is the main topic of this paper, retains all the main features of the maximum interval algorithm. In this paper, we will use the ε − *SVR*, which is a common form of regression estimation, and we will propose an insensitive loss function ε to ignore the error within a certain upper and lower range of the true value, as shown in Figure 6, ξ measures the cost of the error at the training points in linear regression while the error within the insensitive region of ε is zero.

Further, the final single-objective optimization problem with constraints takes the following expression:


(6) $$\begin{array}{r} \text{ minimize }{\left\|w\right\|}^{2}+C\sum_{i=1}^{l}\left(\xi_{i}^{2}+{\hat{\xi }}_{i}^{2}\right),\\ \text{ subject to }\left(\langle w\;\cdot \;x_{i}\rangle +b\right)-y_{i}\leq \varepsilon +\xi_{i},\\ y_{i}-\left(\langle w\;\cdot \;x_{i}\rangle +b\right)\leq \varepsilon +{\hat{\xi }}_{i},\xi_{i},{\hat{\xi }}_{i}\geq 0\;,i=1,\ldots ,l \end{array}$$

The kernel function is a widely used computational tool in SVM (Cai et al., 2019), which can calculate the inner product 〈ϕ(*x*_*i*_)·ϕ (*x*)〉 in the feature space directly, then build a nonlinear learner. In this paper, a Gaussian kernel function is used for high-dimensional mapping, and the equation is as follows:


(7) $$K\langle x_{i},x_{j}\rangle =exp\;(-\frac{{\left\|x_{i}-x_{j}\right\|}^{2}}{2\sigma^{2}})$$

Where σ is the scale of Gaussian kernel function.

### 3.3. Parameter optimization

As with most learning algorithms, the hyperparameters in the SVR model determine the performance of the SVR model, including the regularization parameter C, the insensitivity parameter ε and the radial basis kernel parameter σ (Xiao et al., 2008). For different nonlinear regression problems, it is necessary to select different hyperparameters to find the optimal high-dimensional feature space to reflect the characteristics of the data (Jia et al., 2022).

**Figure 7:**
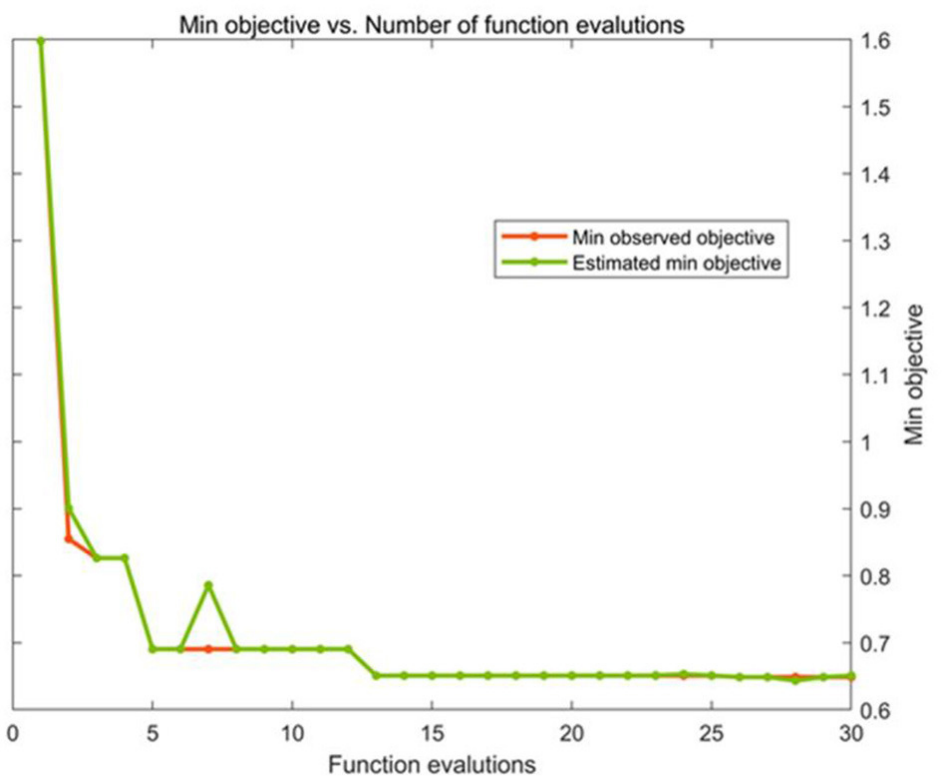
Model parameter optimization process.

K-fold cross-validation is a statistical concept. Its practice is to divide the training set into K equal parts, and take the first part as the validation set and the rest as the training set in the first round. In the second round, take the second part as the verification set, the rest as the training set, and so on. Finally, the average error of K-fold cross validation is calculated to represent the training effect of the model. Bayesian optimization is a common method for tuning parameters in machine learning. The main principle is to perform probabilistic sampling in the feature space and return the optimal solution from the sampling point after multiple iterations. In this paper, we will use Bayesian optimization to perform automatic parameter search to minimize the 5-fold cross validation loss of the SVR model that meets the training set samples. The optimization process of one SVR model is shown in Figure 7.

Optimal parameters of the five SVR models were obtained, which were shown in Table 3.

**Table 3:** Optimal SVR parameters.

**Variable name**	**Regularization parameter C**	**Insensitivity parameter ε**	**Radial basis kernel parameter σ**
Down_Y	50.312	0.075441	6.3182
Left_X	99.542	0.0094612	4.7863
Up_Y	7.0772	0.071628	1.6875
Right_X	0.81525	0.00020469	0.0015695
Balance point	115.43	0.0086606	3.2569

### 3.4. Test set comparison

In this section, the model performance is evaluated using a test set based on the stable region regression model obtained from the training above. The mean square error (MSE) of the test set is selected to represent the relative closeness between the predicted output and the expected output. It is also used to evaluate the generalization ability of the model (Jingxu, 2006). The form of MSE is as follows:


(8) $$MSE=\frac{1}{m}\sum_{i=1}^{m}{\left(Y_{i}-\hat{Y_{i}}\right)}^{2}$$

From the Table 4, we can see that the maximum MSE of the boundary of the sideslip angular velocity is 0.0428 (rad.s^−1^)^2^, while the maximum MSE of the boundary of the sideslip angle is 0.0030 rad^2^, which proves that the stable region regression model has strong generalization ability.

**Table 4:** MSE values for test set.

	**Left_X/rad**	**Down_Y/rad.s^−1^**	**Right_X/rad**	**Up_Y/rad.s^−1^**	**Balance point/rad**
MSE	0.0030	0.0423	0.0028	0.0428	0.0024

## 4. Summary of influential factors & effect analysis

**Figure 8:**
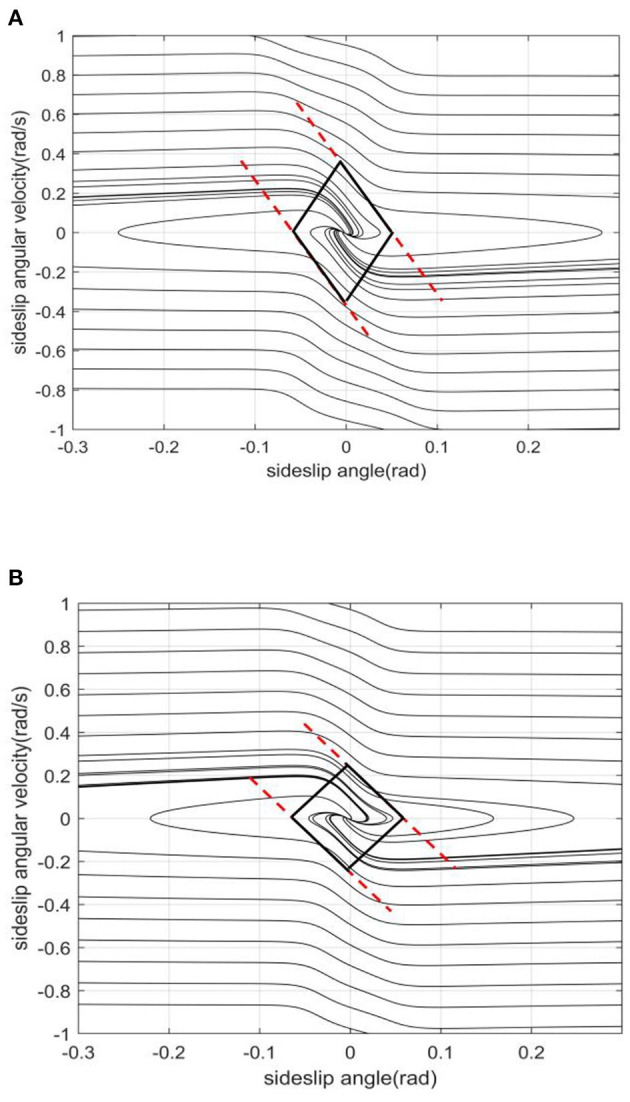
$\left[\mu ,\;\delta ,\;\frac{a}{L}\right]=\left[0.2,\;0,\;0.4\right]$. **(A)** *v* = 20. **(B)** *v* = 30.

**Figure 9:**
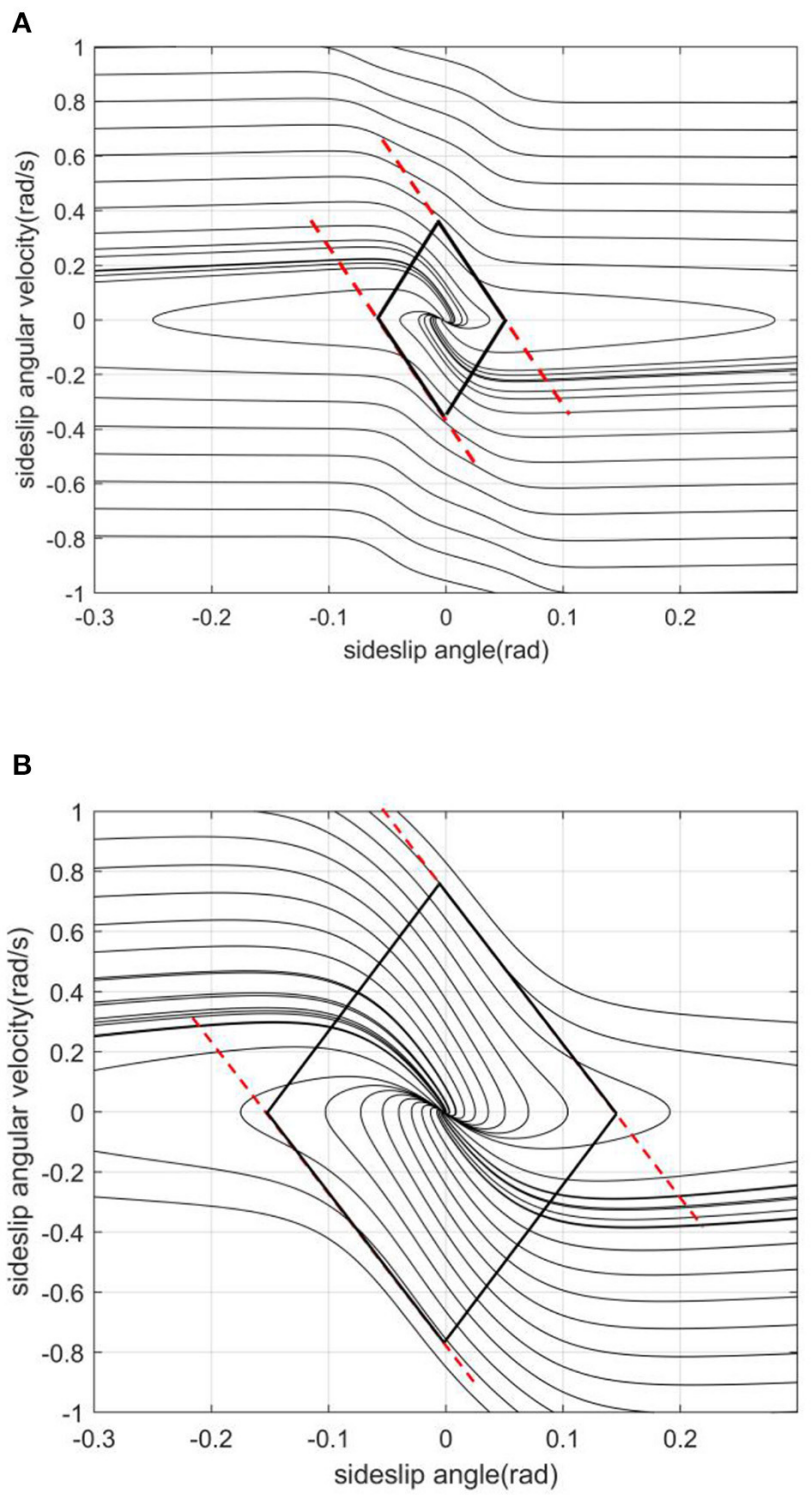
$\left[v,\;\delta ,\;\frac{a}{L}\right]=\left[20,\;0,\;0.4\right]$. **(A)** μ = 0.2. **(B)** μ = 0.6.

**Figure 10:**
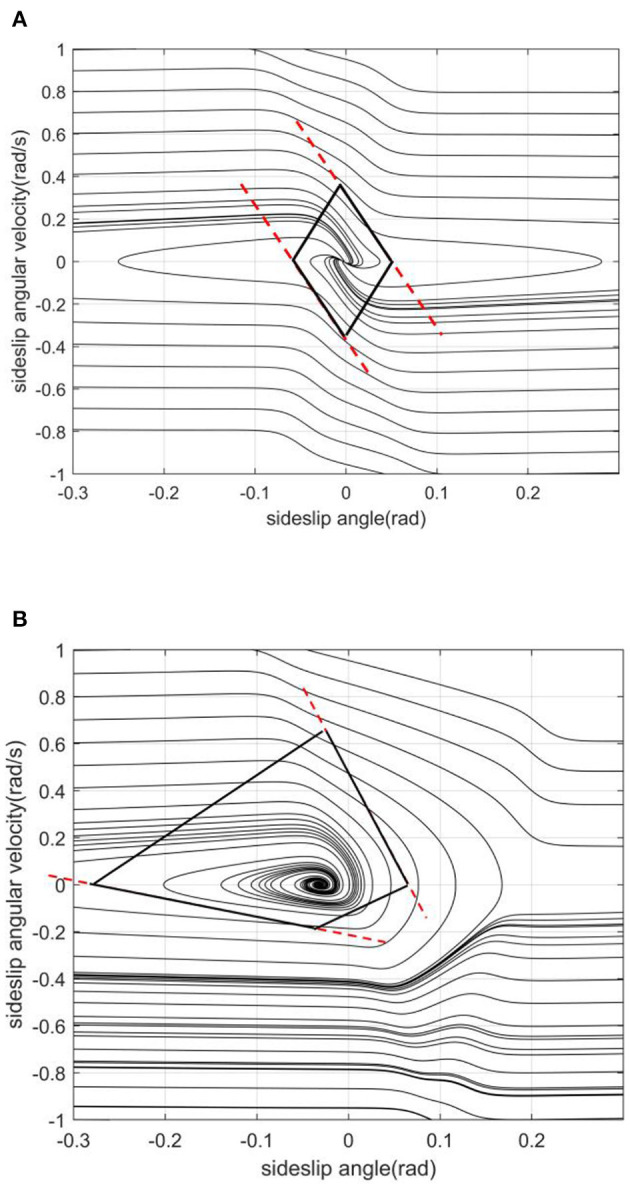
$\left[\mu ,\;v,\;\frac{a}{L}\right]=\left[0.2,\;20,\;0.4\right]$. **(A)** δ = 0. **(B)** δ = 10.

**Figure 11:**
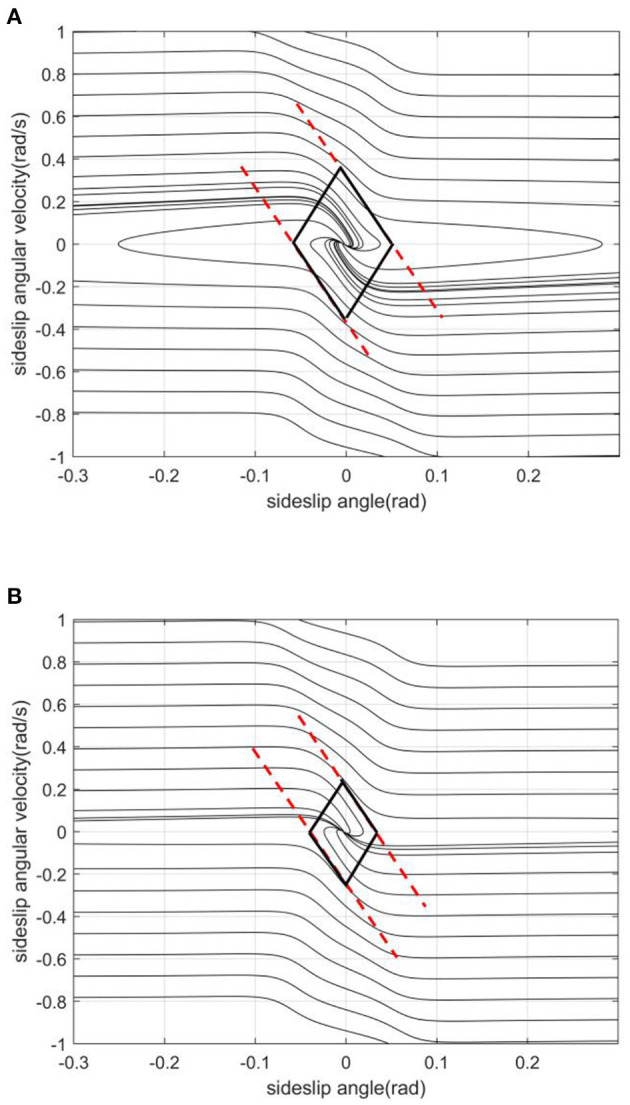
[μ, *v*, δ] = [0.2, 20, 0]. **(A)** *a*/*L* = 0.4. **(B)** *a*/*L* = 0.4881.

According to the SVR model of vehicle stable region, the influencing factors of vehicle stability region are summarized and analyzed. The intercept of the stable region boundary on the horizontal axis characterizes the limit of the stable-state sideslip angle, which is the base point of the whole stable region boundary. The slope of the boundary represents the limit of the sideslip angle under different sideslip angular velocities. The smaller the absolute value of the boundary slope, the stronger the limit of the boundary on the sideslip angle under transient conditions (Huang et al., 2021). Through the analysis of the phase plane stable region and the quadrilateral stability boundary, the following conclusions are drawn:

1. The slope of the left and right boundaries is mainly affected by vehicle speed. In Figure 8, with the increase of vehicle speed, the values of the left and right boundaries remain basically unchanged, and the absolute value of the boundary slope decreases with the increase of vehicle speed. This shows that under the same sideslip angle, the limit of transient sideslip angular velocity increases, the convergent phase trajectory decreases significantly, and the stable region of the phase plane shrinks.
2. The intercept of the stable boundary is mainly affected by the road adhesion coefficient. In Figure 9, with the decrease of the adhesion coefficient, the slope of the non-adjacent boundary of the stable region remains basically unchanged, but the left and right boundary values converge to the equilibrium point. This shows that under the same sideslip angle, the restriction on the transient sideslip angle is strengthened, the stable region shrinks, and the trajectory of convergence in the phase plane decreases.
3. The effect of the front wheel angle on the phase plane mainly is making the phase trajectory no longer symmetrical. In Figure 10, when the current wheel angle is small, the number of convergence trajectories does not change significantly, but the stable region will flatten along the horizontal axis, and the asymmetry is not obvious at this time. When the current wheel angle is large, the slope of the left boundary will change so that it is no longer parallel to the right line. This means that the absolute value of the slope decreases, resulting in a greater limit of the steady-state sideslip angle and a sharp reduction of the transient sideslip angle limit.
4. The main effect of the centroid position on the stable region is the distance from the centroid position to the front and rear axles. In the Figure 11, when the centroid position is shifted backward, the slope of the boundary is basically unchanged, the absolute value of the intercept between the left and right boundaries of the stable region decreases, the convergent phase trajectory is significantly reduced, and the stable region of the phase plane decreases.

## 5. Stability controller designing and simulation test

### 5.1. Stability controller designing

When there is a high risk of instability, the ESP system will automatically intervene to prevent the vehicle from losing control. Both DYC and Active Front Steering (AFS) control technology can improve the driving stability of the vehicle. Among them, DYC generates a transverse torque acting on the body through four-wheel differential braking force to achieve vehicle stability control. It is widely used because of its good performance in vehicle handling and trajectory keeping.

Based on the following 2-DOF differential equation of vehicle motion, vehicle stability is closely related to vehicle state:


(9) $$\left\{ \begin{array}{l} \sum_{i=1}^{4}F_{Yi}=m\;(\dot{v_{y}}+v_{x}r)\\ \;M_{z}=I_{z}\dot{r} \end{array}\right.$$

Considering the influence of deceleration caused by four-wheel brake distribution on vehicle speed, vehicle speed is considered as a time-varying state quantity. The expression of sideslip angular velocity is as follows.


(10) $$\dot{\beta }=\frac{{\dot{v}}_{y}}{v_{x}}-\frac{\dot{v_{x}}}{v_{x}}\beta$$

State space equation belongs to the system formula of modern control theory. It starts from the differential equation of the system and introduces the concepts of system state, input and output to construct the system expression. The following vehicle stability state space equation is obtained by further simplification:


(11) $$\begin{array}{l} \left[\begin{array}{c} \dot{r}\\ \dot{\beta } \end{array}\right]=\left[\begin{array}{cc} \frac{a^{2}k_{f}+b^{2}k_{r}}{v_{x}I_{z}}\; & \frac{ak_{f}-bk_{r}}{I_{z}}\\ \frac{ak_{f}-bk_{r}}{mv_{x}^{2}}-1\; & \frac{k_{f}+k_{r}}{mv_{x}}-\frac{\dot{v_{x}}}{v_{x}} \end{array}\right]\left[\begin{array}{c} r\\ \beta \end{array}\right]+\left[\begin{array}{c} \frac{1}{I_{z}}\\ 0 \end{array}\right]\\ \;\Delta T+\left[\begin{array}{c} -\frac{ak_{f}}{I_{z}}\\ -\frac{k_{f}}{mv_{x}} \end{array}\right]\delta \end{array}$$

Where *a*, *b* is the distance from the center of mass to the front and rear axles, *k*_*f*_, *k*_*r*_ is the lateral stiffness of front and rear axle, ΔT is the additional yaw moment.

**Figure 12:**
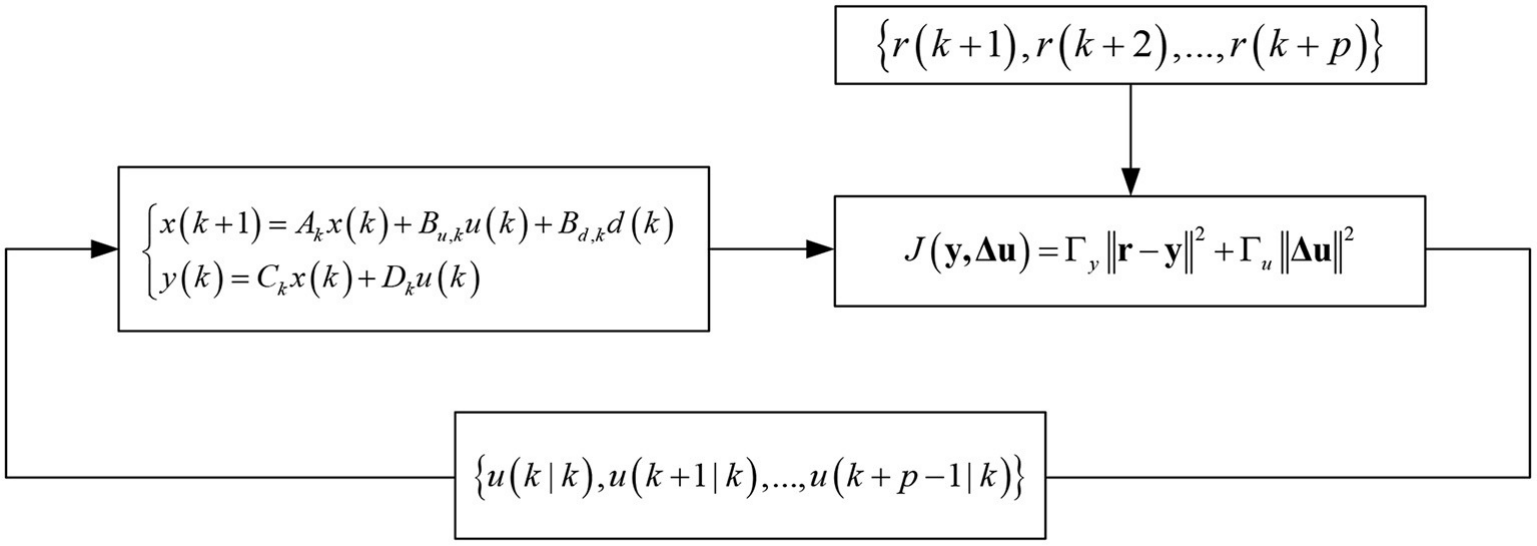
LTV-MPC open-loop optimal solution.

Model predictive control (MPC) is one of the model-based feedback control strategies, which is widely used in various control systems because of its good control effect and robustness. The basic principle of MPC can be summarized as follows: at each sampling time, update the optimization problem with the latest measured value, solve the updated open-loop optimization problem and apply the first component of the optimization solution u^*^ (*k*|*k*) to the system. LTV-MPC is an extended control method of MPC. Because MPC is an algorithm with high requirements for model accuracy, its control accuracy will decline when the system status is updated. However, LTV-MPC considers the state change of the linear control system and has stronger adaptability for time-varying systems. Figure 12 shows the schematic diagram of the open-loop optimal solution of LTV-MPC.

Considering the time-varying of the vehicle system, this paper will establish a DYC stability controller based on LTV-MPC, which includes an upper controller and a lower controller. The upper controller receives the signal from the stability judgment module and gives the expected additional yaw moment at the current moment through the online open-loop solution. The lower controller realizes direct yaw torque control through four-wheel braking force distribution, thus maintaining the stability of the vehicle.

The upper controller is mainly composed of LTV-MPC, while the lower controller is responsible for the four-wheel braking force distribution of the yaw moment, which is adjusted in real time by giving the desired additional yaw moment and desired deceleration speed from the upper controller. The four-wheel braking force needs to satisfy the following constraints.


(12) $$\{\begin{array}{c} ma_{aim}=F_{X1}+F_{X2}+F_{X3}+F_{X4}\\ \Delta T=\left(F_{X2}+F_{X4}\right)L_{a}-\left(F_{X1}+F_{X3}\right)L_{a} \end{array}$$

Where, *F*_*X*1_, *F*_*X*3_, *F*_*X*2_, *F*_*X*4_ corresponds to the left front, left rear, right front, and right rear wheels respectively, *L*_*a*_ is the front and rear axle half axle length, and *a*_*aim*_ is the desired braking deceleration, is given by another upper speed controller, which is not the focus of this paper and will not be discussed more here.

Due to the axle load transfer during braking, the front wheels are subjected to greater vertical loads than the rear wheels, and are subjected to greater braking forces, with the following constraints.


(13) $$\{\begin{array}{c} F_{X3}=\frac{l_{f}g+a_{aim}h_{g}}{l_{r}g-a_{aim}h_{g}}F_{X1}\\ F_{X4}=\frac{l_{f}g+a_{aim}h_{g}\;}{l_{r}g-a_{aim}h_{g}}F_{X2} \end{array}$$

Where *h*_*g*_ is the height of the center of mass of the vehicle.

According to the above constraints, there is a unique four-wheel braking force distribution scheme for the given expected additional yaw moment and expected deceleration. At this point, a DYC stability controller based on LTV-MPC is proposed, and the model-in-loop simulation test will be launched based on the algorithm proposed in the previous section.

### 5.2. Model-in-the-loop simulation testing

**Figure 13:**
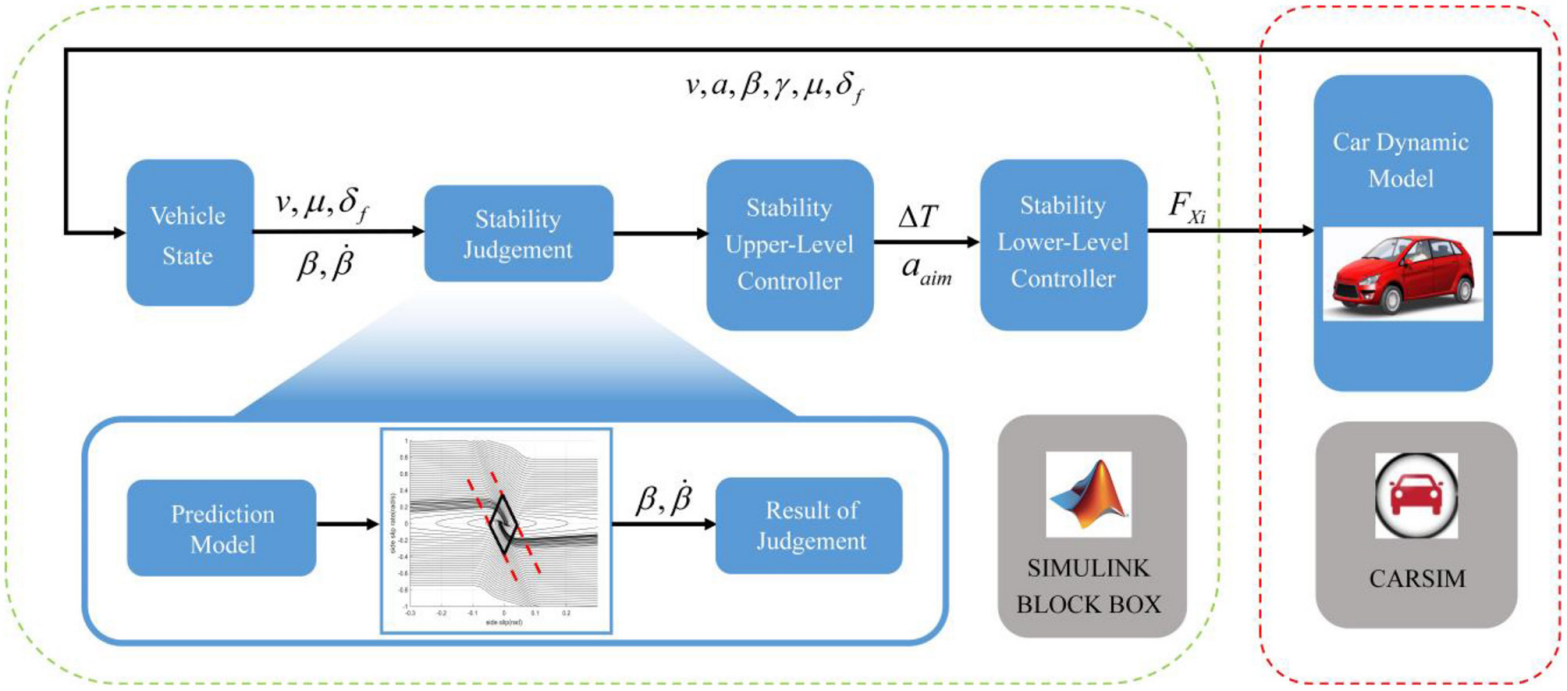
Simulation test architecture.

CARSIM-SIMULINK co-simulation platform is used for model in-loop simulation test. We configured the vehicle dynamics model and operation scenario in CARSIM, and designed the controller and algorithm in SIMULINK (Cong et al., 2022). The scenario set in CARSIM selects a road with different adhesion coefficients between the left and right wheel surfaces: 1.0 on the left and 0.2 on the right. Under such bad road conditions, the stability controller proposed in this paper will work to avoid the instability of vehicle. However, the braking force distribution will lead to the underutilization of the road adhesion coefficient, which will lead to the deterioration of the vehicle braking performance. Therefore, it is necessary to set up a decision module to determine the current stability state of the vehicle and decide whether to apply stability control (Guo et al., 2018). The stability decision module is given by the dynamic stable region regression model proposed in this paper, as shown in Figure 13. According to the vehicle state input, the stable region regression model gives the vehicle driving stable region on the phase diagram of the sideslip angle—sideslip angular velocity, as the criterion of vehicle stability.

**Figure 14:**
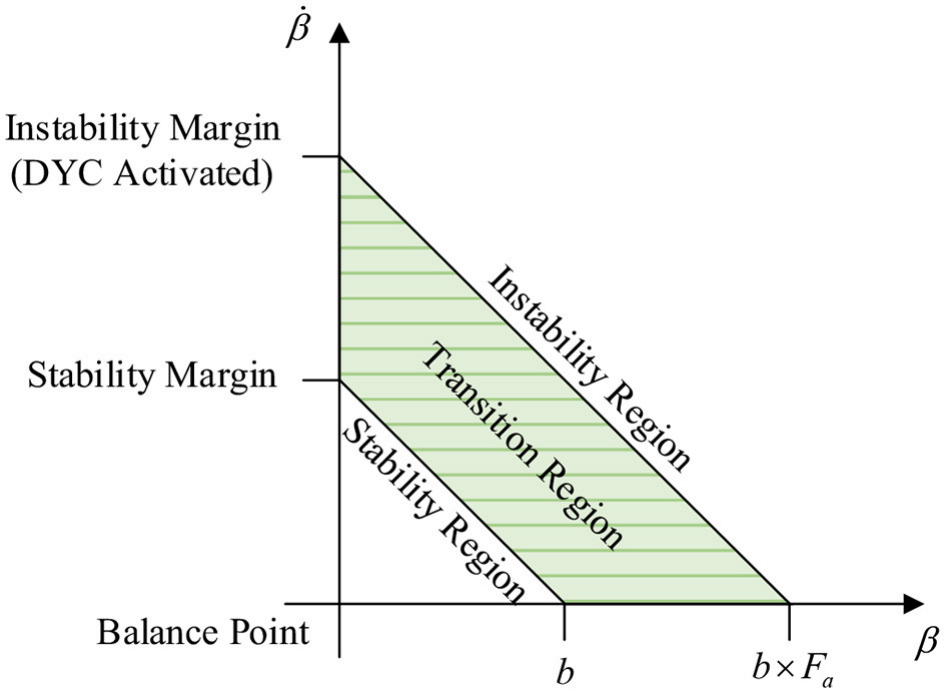
Stable transition area.

Since the artificial division of stable region is strict, so in the stable region, we regard that the vehicle is in the absolute stability state. At the same time, in order to prevent the controller from switching frequently and repeatedly, we set a relaxation factor *F*_*a*_ which is greater than 1. Multiply each boundary of the stable region by *F*_*a*_ to obtain the unstable boundary. The vehicle state outside the unstable boundary is regarded as unstable, and the middle of the unstable boundary and the stable boundary is regarded as the transition region. As shown in Figure 14.

On this basis, the test process of stability decision and control algorithm is proposed. The following is a model-in-the-loop simulation test method to compare the traditional AEB, the equipment of stability control (SC) without stability judgment decision (SVR) and the equipment of SC and SVR, we use the vehicle sideslip angle and the obstacle distance to measure the stability safety performance and braking safety performance of each scheme, which can verify the feasibility of the vehicle stability control strategy (Zhang et al., 2016; Wu et al., 2022).

**Figure 15:**
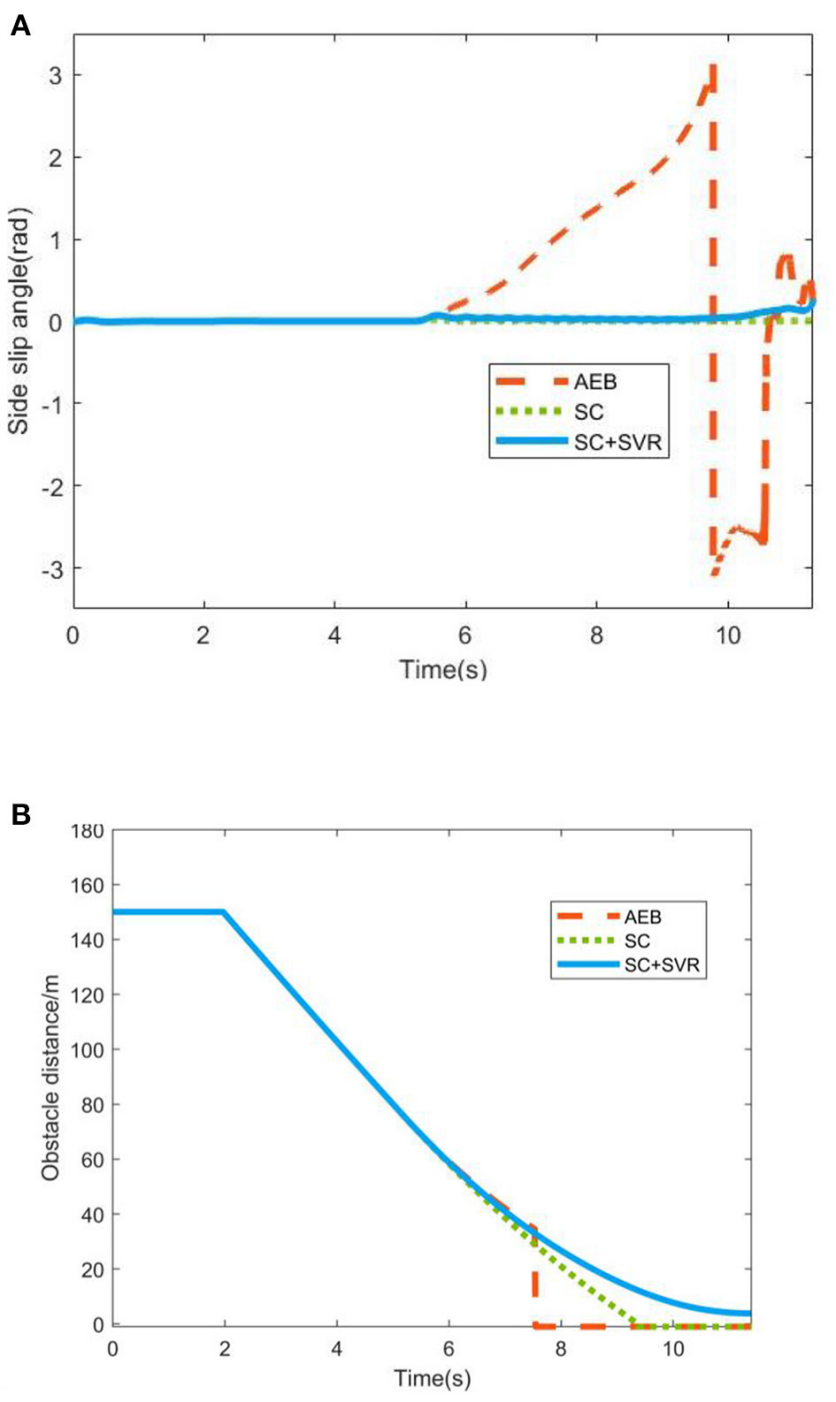
Simulation results. **(A)** The sideslip angle of three working conditions. **(B)** The obstacle distance of three working conditions.

By adjusting and calibrating the *F*_*a*_, the simulation results were obtained as shown in Figure 15.

From the simulation test results, for emergency braking under bad road conditions, the traditional AEB scheme has a serious instability and side slip (Wu, 2007), resulting in the sensor not being able to recognize the obstacle in front, which obviously does not meet the requirements of stability safety. The scheme of adding SC without SVR can ensure the stability safety of the vehicle, but due to the excessive intervention of the stability controller, the braking safety of the vehicle cannot meet the requirements. The scheme of adding SC and SVR can not only ensure that the vehicle stops at a safe distance in front of the obstacle, but also ensure that the sideslip angle is within the acceptable range. In summary, the rationality of using the stable region regression model proposed in this paper for stability judgment is verified, and the efficiency of the LTV-MPC stability control algorithm proposed in this paper is illustrated.

## 6. Conclusions & outlooks

The algorithm proposed by this paper is mainly applied to scenarios where lateral instability of the vehicle may occur. In another word, the algorithm is used to determine the stability state of the vehicle, and avoid the occurrence of vehicle instability. The conclusions are as follows:

1. Based on the traditional double-line method, this paper proposes an improved double-line method for quadrilateral stable region, and carries out a large amount of work on stable region dividing to establish a sample data set for supervised learning training and testing.
2. In this paper, an SVR-based dynamic stable region regression model is proposed based on the vehicle $\beta -\dot{\beta }\;$phase plane to provide a criterion for the real-time stability of vehicle driving. The result of the test set indicates that this dynamic stable region regression model has a strong generalization ability.
3. In this paper, we extend the regression model of dynamic stable region, consider the real-time input of vehicle state, and the causal analysis of important factors and vehicle driving stable region is performed and summarized.
4. In this paper, based on LTV-MPC, a DYC stability controller is carried out, and the algorithm verification process is designed. Compared with the traditional AEB scheme and the SC-equipped scheme, the SC&SVR can coordinate the braking safety and stability safety of the vehicle. The result verifies the rationality of using the stable region regression model proposed in this paper for stability evaluation, as well as the efficiency of the stability control algorithm of the DYC stability controller.

However, there are still many limitations of the algorithm.

1. Due to the use of pre-calibrated data sets, when vehicle parameters change a lot, the accuracy of the model will be reduced.
2. The test vehicle needs to have four-wheel differential braking capability.
3. The adaptive MPC algorithm consumes a lot of arithmetic power and requires high accuracy of the model, so it needs to be used with a better performance observer for actual testing.

## Data availability statement

The original contributions presented in the study are included in the article/supplementary material, further inquiries can be directed to the corresponding author.

## Author contributions

All authors listed have made a substantial, direct, and intellectual contribution to the work and approved it for publication.
